# Comparison of volume of the forebrain, subarachnoid space and lateral ventricles between dogs with idiopathic epilepsy and controls using a stereological approach: Cavalieri’s principle

**DOI:** 10.1186/s40575-021-00101-6

**Published:** 2021-03-10

**Authors:** Fraje Watson, A. Augusto Coppi, Holger A. Volk, Rowena M. A. Packer, Anna Tauro, Clare Rusbridge

**Affiliations:** 1 Royal Veterinary College, Hawkshead Lane, Hertfordshire, Hatfield AL9 7TA UK; 2 Present Address: University College London, Division of Surgery & Interventional Science, Aspire CREATe, Royal National Orthopaedic Hospital, Brockley Hill, Stanmore, Middlesex, HA7 4LP UK; 3 School of Animal, Rural and Environmental Sciences, Nottingham Trent University, Nottingham, NG25 0QF UK; 4 Department of Small Animal Medicine and Surgery, University of Veterinary Medicine Hannover, Bünteweg, 30559 Hanover, Germany; 5 Chestergates Veterinary Specialists, Telford Court, Units E and F, Gates Lane, Chester, CH1 6LT UK; 6 School of Veterinary Medicine, Faculty of Healthy & Medical Sciences, University of Surrey, Main Academic Building (VSM), Daphne Jackson Road, Guildford, Surrey, GU2 7AL UK

**Keywords:** Canine, Idiopathic epilepsy, Cavalieri principle, Design-based stereology, Brain volume, Forebrain, Subarachnoid space, Lateral ventricles

## Abstract

**Background:**

Canine idiopathic epilepsy (IE) is the most common chronic neurological brain disease in dogs, yet it can only be diagnosed by exclusion of all other potential causes. In people, epilepsy has been associated with a reduction in brain volume. The objective was to estimate the volume of the forebrain (FB), subarachnoid space (SAS) and lateral ventricles (LV) in dogs with IE compared to controls using Cavalieri’s principle. MRI scans of case and control dogs were identified from two neurology referral hospital databases. Eight breeds with increased odds of having IE were included: Golden Retriever, Labrador Retriever, Cocker Spaniel, Border terrier, German Shepherd dog, Parson Jack Russell terrier, Boxer, and Border Collie. Five dogs of each breed with IE and up to five controls were systematically and uniformly randomly sampled (SURS). The volume of the FB, SAS and LV were estimated from MRI scans by one blinded observer using Cavalieri’s principle.

**Results:**

One hundred-two dogs were identified; 56 were diagnosed with IE and 46 were controls. There was no statistically significant difference in FB, SAS and LV volume between dogs with IE and controls. Dogs with a history of status epilepticus had significantly larger FB than those without (*p* = 0.05). There was a border-line trend for LV volume to increase with increasing length of seizure history in the IE group (*p* = 0.055).

**Conclusion:**

The volumes of the FB, SAS and LV are not different between dogs with IE and controls, so IE remains a diagnosis of exclusion with no specific neuroanatomical biomarkers identified. This is the first time FB and SAS volume has been compared in dogs with IE. Unfortunately, we have shown that the results reporting significantly larger FBs in dogs with status epilepticus and LV volume increase with length of seizure history were likely confounded by breed and should be interpreted cautiously. Whilst these associations are interesting and clinically relevant, further investigation with breed-specific or larger, breed-diverse populations are required to permit strong conclusions. The Cavalieri principle provided an effective estimation of FB, SAS and LV volumes on MRI, but may be too time-intensive for use in clinical practice.

## Background

Idiopathic epilepsy (IE) is estimated to affect between 0.60–0.75% of the general canine population [1, 2]. Unlike structural epilepsy or reactive seizures, where a cause can be found with rigorous clinical investigations, the diagnosis of IE remains one of exclusion [3]. Magnetic Resonance Imaging (MRI) forms part of the Tier 2 confidence level of diagnostic certainty of IE recommendations made by the International Veterinary Epilepsy Task Force (IVETF), and is a common diagnostic tool in veterinary referral hospitals [3].

Research in people with epilepsy has uncovered a vast range of differences in brain volume of different anatomical structures compared to controls, such as the lateral ventricles (LV), hippocampus and thalamus, amongst others [4–13]. Increased volume or asymmetry of the LV have been associated with psychiatric and neurological conditions in people with epilepsy [12]. Interestingly, in people with temporal lobe epilepsy and idiopathic generalised epilepsy, significant brain atrophy has been reported compared to healthy controls when measured an average of 17.90 years after the onset of epilepsy, which could be the result of repeated seizures over time [5]. Unfortunately, the cross-sectional nature of this study may give rise to conclusions of causality resulting from primary abnormalities or secondary changes..

Veterinary volumetric studies have started to outline normal ranges for neuroanatomical structures of neurologically normal patients using MRI [14], however, target areas are still limited and additional studies are required to broaden the data available. This is particularly challenging given the conformational diversity of the dog [15], which is one of the most phenotypically diverse species on earth especially for the shape of the skull and cranial cavity [16]. In addition, automated voxel-based 2D morphometry techniques available in human medicine have only recently emerged in veterinary medicine [17, 18], meaning that in most instances copious time and practice is essential to take such measurements [19]. Commonly used methods in veterinary medicine include: estimations based on area width, length and depth [20, 21], volume rendering via slice-by-slice image segmentation [22] and manual pixel selection [22].

Similarly to human studies [12], many veterinary studies to date have focused on the volume or asymmetry of the lateral ventricles (LV) and have largely reported either no difference in total LV volume or left-right asymmetry in dogs with IE compared to controls [21, 23–27]. One veterinary study [28] found evidence of hippocampal atrophy in dogs with IE compared to controls whilst another found reduced grey matter volume in research beagles with and without spontaneous IE [17]. Most studies tentatively assume that these changes are incidental rather than evidence of pathology related to IE. Certainly, a hinderance in veterinary medicine is the expensive specialist software and time required for a clinician or researcher to individually map and estimate the volume of the anatomical structures of interest. Research in this area could be advanced by the discovery of a practical and time-saving alternative technique.

The objective of this study was to assess the practicality and ease of a design-based stereological method for volume estimation, the Cavalieri principle, and its use to estimate and compare the volume of the FB, SAS and LV in dogs with IE compared to controls. We hypothesise that the volume of the FB, SAS and LV will be different in dogs with IE compared to controls. We also hypothesise that these volumes will be associated with other clinical variables in the sub-population of dogs with IE, particularly with seizure history. Additionally, we will compare sub-groups of dogs with IE to compare volume of FB, SAS and LV in dogs with IE who experience cluster seizures (CS) and status epilepticus (SE) to dogs with IE who do not.

## Results

One hundred-two dogs were identified; 56 were diagnosed with IE per the criteria above and 46 acted as controls. Forty-nine dogs were seen at Fitzpatrick Referrals Orthopaedics & Neurology, UK (48.04%; 26 cases and 23 controls), and 53 dogs were seen at the Queen Mother Hospital for Animals, UK (51.96%; 30 cases and 23 controls). This consisted of 37 Labrador Retrievers (36.27%), 15 Golden Retrievers (14.71%), 15 German Shepherds (14.71%), 15 Border Collies (14.71%), 13 Cocker Spaniels (12.75%), three Border terriers (2.94%), three Boxers (2.94%), and one Parsons Jack Russell terrier (0.98%). Table 1 reports the clinical diagnosis of control dogs. Neither age nor body weight were significantly different between dogs with IE and controls (Table 2). Mean MRI slice thickness was 3.79 mm (SD = 0.41) and mean slice interval was 22.58% (0.86 mm) (SD = 0.06) of the slice thickness.

**Table 1:** Clinical diagnosis of control group dogs

Breed	Diagnosis (frequency)
Golden Retriever	Anxiety attacks (2), Benign resting tremor (2), Degenerative spinal cord disorder (2), Foreign body-associated abscess in the masticatory muscle (2), Idiopathic facial and vestibular neuritis (2)
Border Collie	Immune-mediated CNS inflammatory disease (2), Myelodysplasia (2), Retro-bulbar/rostral mandibular abscess (2), Behavioural disorder (2)
Border Terrier	T12–13 high-velocity low volume disc (2), facial pain (2)
Boxer	Idiopathic facial nerve paralysis and peripheral vestibular syndrome (2), Pain response (2)
Cocker Spaniel	Idiopathic facial paralysis (2), Idiopathic facial nerve paralysis and peripheral vestibular syndrome (2), Idiopathic Horner’s (2), Idiopathic vestibular syndrome (2), Intermittent abnormal right thoracic limb proprioception (2), Episodic gait abnormality (2), Toxin exposure (2), Atlanto-axial subluxation (2), Behavioural disorder (3), Cerebellar disease (2)
German Shepherd Dog	Idiopathic Horner’s and congenital nasal dermoid sinus (2), Inflammatory polycranial neuropathy of infectious origin (2), Juvenile degenerative myelopathy (2), Lumbosacral disease (3), Metronidazole toxicity (2)
Labrador	Idiopathic head tremors (2), Idiopathic peripheral vestibular syndrome (2), Idiopathic trigeminal neuritis and Horner’s syndrome (2), Idiopathic trigeminal neuropathy (2), Idiopathic vestibular syndrome (2), Intermittent cerebellar ataxia (2), Optic neuritis (2), Metronidazole toxicity (2), Neuromuscular disease (2), Osteosarcoma spine and pelvic limb (2), Paroxysmal dyskinesia or movement disorder (2), Wobblers (2), Abnormal behaviour (2), Collapse/vestibular episodes (2), Extraocular myositis (2), Hypothyroidism (2)

**Table 2:** Descriptive data of idiopathic epilepsy and control group dogs

	Idiopathic Epilepsy	Control	All
**Age (months)**	50.05 (SD = 19.32)	45.20 (SD = 25.72)	47.86 (SD = 22.45)
**Body weight (kg)**	29.41 (SD = 8.62)	27.69 (SD = 9.77)	28.72 (SD = 9.09)
**Male**	42	28	70
*Entire*	*15*	*10*	*25*
*Neutered*	*27*	*18*	*45*
**Female**	14	18	32
*Entire*	*6*	*7*	*13*
*Neutered*	*8*	*11*	*19*
**Breed**			
*Golden Retriever*	10	5	15
*Border Collie*	11	4	15
*Border Terrier*	1	2	3
*Boxer*	1	2	3
*Cocker Spaniel*	2	11	13
*German Shepherd*	9	6	15
*Labrador Retriever*	21	16	37
*Parson’s Jack Russell Terrier*	1	0	1

All dogs with IE had a clinically normal inter-ictal neurological examination, haematology and biochemistry and an unremarkable brain on MRI. In addition to this, tests to give further confidence in diagnosis were done for 50 of the IE dogs (89.29%); 26 dogs also had both cerebrospinal fluid (CSF) and Bile Acid Stimulation Test (BAST) (46.43%), 20 dogs had CSF analysis (35.71%), and four dogs had BAST (7.14%). Two dogs had mildly elevated protein levels in their CSF, potentially associated with a recent seizure.

Dogs with IE had a mean inter-ictal period of 31.16 days (SD = 34.21) and, prior to the MRI scan, last had a seizure a mean of 16.46 days ago (SD = 16.61). At the time of MRI scan 27 dogs (51.79%) were drug-naïve, 20 (35.71%) were receiving one anti-epileptic drug (AED), eight (14.29%) were receiving two AEDs and one dog (1.79%) was receiving three AEDs.

### Inter-observer agreement

Throughout training and the pilot study, measurements were taken in cm^3^ and retrospectively converted to mm^3^. The intra-class coefficient was 0.948, 0.936 and 0.977 for the FB, LV and SAS, respectively. Agreement was considered excellent for all three anatomic areas. A mean of 70.80, 70.20 and 85.80 points were counted within each region of interest for the FB, LV and SAS, respectively. This highlighted the need for a denser grid to ensure at least 200 points were counted per region of interest [29].

### Effect of IE

**Fig. 1:**
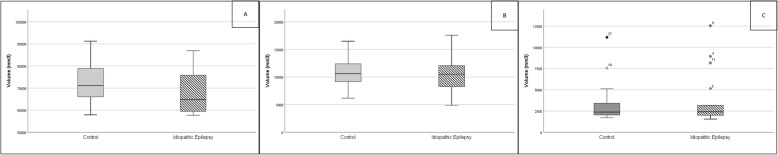
Boxplot graphs for volumes of the forebrain **a**, subarachnoid space **b** and lateral ventricles **c** for control dogs (grey) and dogs with Idiopathic Epilepsy (striped)

Analyses included 37 randomly selected Labradors, Golden Retrievers, German Shepherd Dogs and Border Collies; 17 with IE and 20 controls. Mean FB volume for dogs with IE was 68,714.38mm^3^ (CV = 13.90; CE = 0.01) and 72,270.13mm^3^ (CV = 13.07; CE = 0.01) for controls. Mean SAS volume for dogs with IE was 10,491.44mm^3^ (CV = 27.19; CE = 0.04) and 10,756.49mm^3^ (CV = 25.39; CE = 0.02) for controls. Mean LV volume for dogs with IE was 3677.65mm^3^ (CV = 82.46; CE = 0.09) and 3319.53mm^3^ (CV = 71.70; CE = 0.05) for controls. There was no statistically significant difference in FB (*p* = 0.218), SAS (*p* = 0.828) and LV (*p* = 0.869) volume between dogs with IE and controls (Fig. 1). A mean of 3291, 473 and 162 points were counted for each brain region, FB, SAS and LV, respectively.

### Effect of time since IE onset and LV volume

**Fig. 2:**
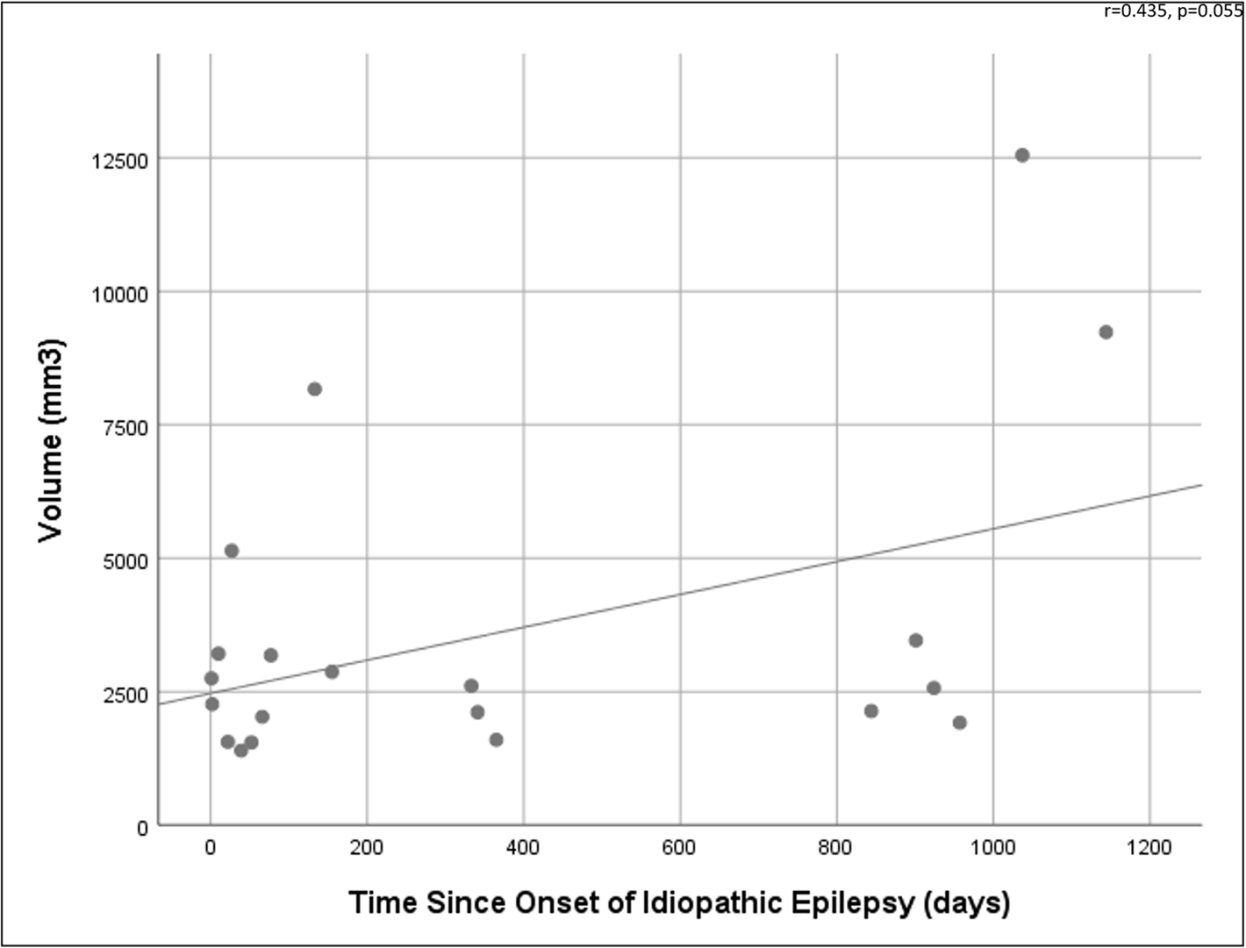
Scatterplot graph shows the relationship between volume of the lateral ventricles and the number of days since onset of idiopathic epilepsy

Time since onset of IE was available for 20 dogs. Mean time since onset of IE was 371.50 days (SD = 418.99 days). There was a trend towards LV volume increasing with the length of seizure history (r = 0.435, *p* = 0.055) (Fig. 2).

### Cluster seizures

Twenty-one dogs were reported to have experienced CS, the remaining 37 were IE-controls (i.e. dogs diagnosed with IE with no history of CS) (Table 3). Five CS dogs and 5 IE-controls were randomly selected including Labradors, Golden Retrievers, German Shepherd Dogs and Border Collies. There were no significant differences between age or body weight for CS dogs and IE-controls. Mean FB volume was 68,747.88mm^3^ (CV = 14.44, CE = 0.10) for dogs with CS and 70,933.32mm^3^ (CV = 16.00,CE = 0.01) for IE-controls. Mean LV volume was 2465.69mm^3^ (CV = 33.39, CE = 0.05) for dogs with CS and 2546.86mm^3^ (CV = 19.16, CE = 0.05) for IE-controls. Mean SAS volume was 10,714.21mm^3^ (CV = 51.87, CE = 0.02) for dogs with CS and 9146.33mm^3^ (CV = 34.23, CE = 0.02) for IE-controls. There was no significant difference in FB (*p* = 0.754), SAS (*p* = 0.598) or LV (*p* = 0.854) volume between groups.

**Table 3:** Descriptive data for cluster seizure and idiopathic epilepsy control group dogs

	Cluster Seizure	Idiopathic Epilepsy Control
**Age (months)**	46.37 (SD = 17.16)	52.28 (SD = 19.75)
**Body weight (kg)**	30.12 (SD = 7.69)	28.98 (SD = 8.93)
**Male**	16 (76.19%)	27 (72.97%)
*Entire*	*6*	*10*
*Neutered*	*10*	*17*
**Female**	5 (23.81%)	10 (27.03%)
*Entire*	*2*	*4*
*Neutered*	*3*	*6*
**Breed**		
*Golden Retriever*	5	6
*Border Collie*	6	4
*Border Terrier*	0	1
*Boxer*	0	1
*Cocker Spaniel*	0	2
*German Shepherd*	6	4
*Labrador Retriever*	4	17
*Parson’s Jack Russell Terrier*	0	1

### Status Epilepticus

**Fig. 3:**
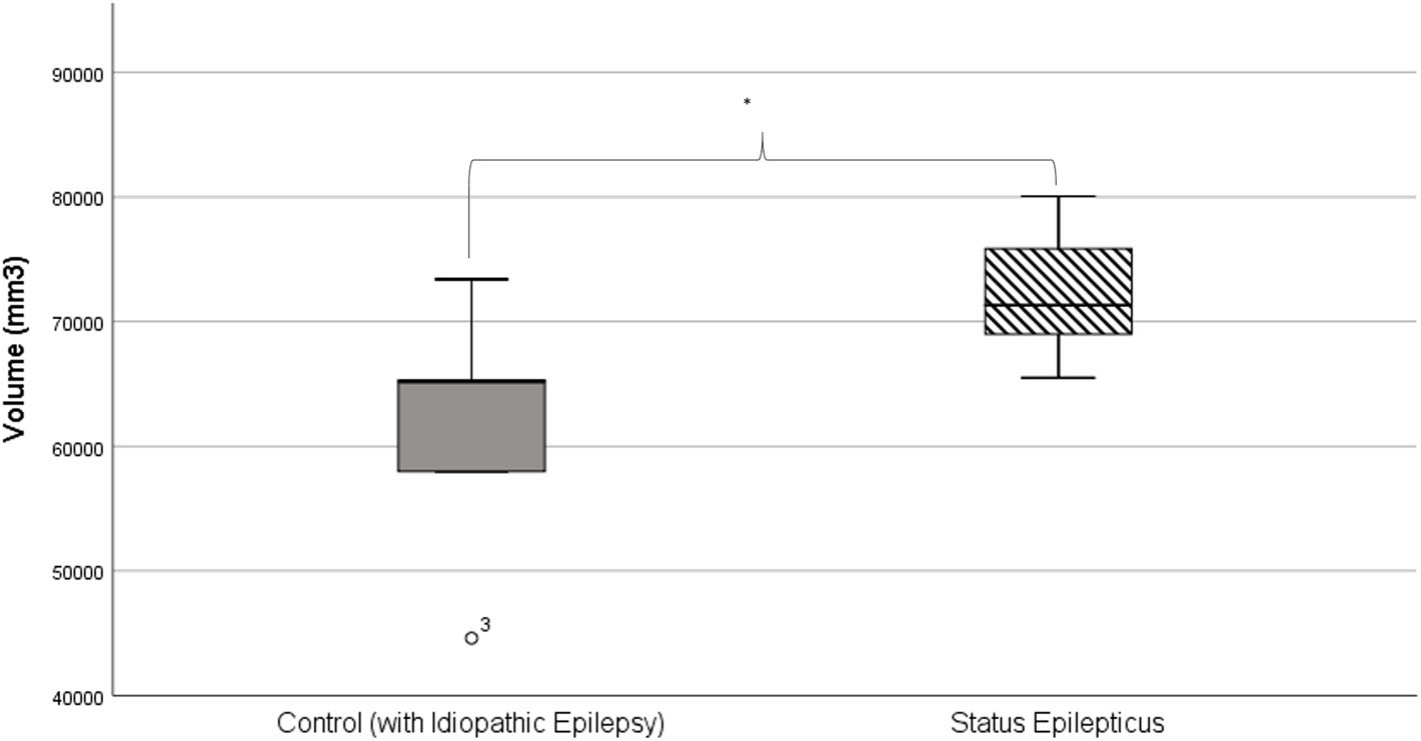
Boxplot graph shows the volume of the forebrain for control dogs with Idiopathic Epilepsy (grey) and dogs with Idiopathic Epilepsy who experience status epilepticus (striped)

Nine dogs were reported to have experienced SE, the remaining 49 were IE-controls (i.e. dogs diagnosed with IE with no history of SE). Descriptive data can be found in Table 4. Five SE dogs and 5 IE-controls were randomly selected including; Labradors, Golden Retrievers, German Shepherd Dogs and Border Collies. There were no significant differences between age or body weight for SE dogs and IE-controls. Mean FB volume was 72,345.62mm^3^ (CV = 7.91, CE = 0.01) for dogs with SE and 61,307.26mm^3^ (CV = 17.62, CE = 0.01) for IE-controls. Mean LV volume was 5062.18mm^3^ (CV = 85.40, CE = 0.04) for dogs with SE and 3508.40mm^3^ (CV = 92.87, CE = 0.04) for IE-controls. Mean SAS volume was 11,071.22mm^3^ (CV = 26.77, CE = 0.01) for dogs with SE and 10,197.66mm^3^ (CV = 55.83, CE = 0.02) for IE-controls. The FB volume of SE dogs was significantly larger than controls (*p* = 0.047) (Fig. 3). There was no significant difference in SAS (*p* = 0.527) or LV (*p* = 0.527) volume between groups.

**Table 4:** Descriptive data for status epilepticus and idiopathic epilepsy control group dogs

	Status Epilepticus	Idiopathic Epilepsy Control
**Age (months)**	51.63 (SD = 12.68)	50.00 (SD = 19.92)
**Body weight (kg)**	33.01 (SD = 8.95)	28.76 (SD = 8.95)
**Male**	9 (100.00%)	34 (69.39%)
*Entire*	*6*	*10*
*Neutered*	*3*	*24*
**Female**	0 (0.00%)	15 (30.61%)
*Entire*	*0*	*6*
*Neutered*	*0*	*9*
**Breed**		
*Golden Retriever*	1	10
*Border Collie*	0	11
*Border Terrier*	0	1
*Boxer*	0	1
*Cocker Spaniel*	0	2
*German Shepherd*	1	9
*Labrador Retriever*	7	14
*Parson’s Jack Russell Terrier*	0	1

**Fig. 4:**
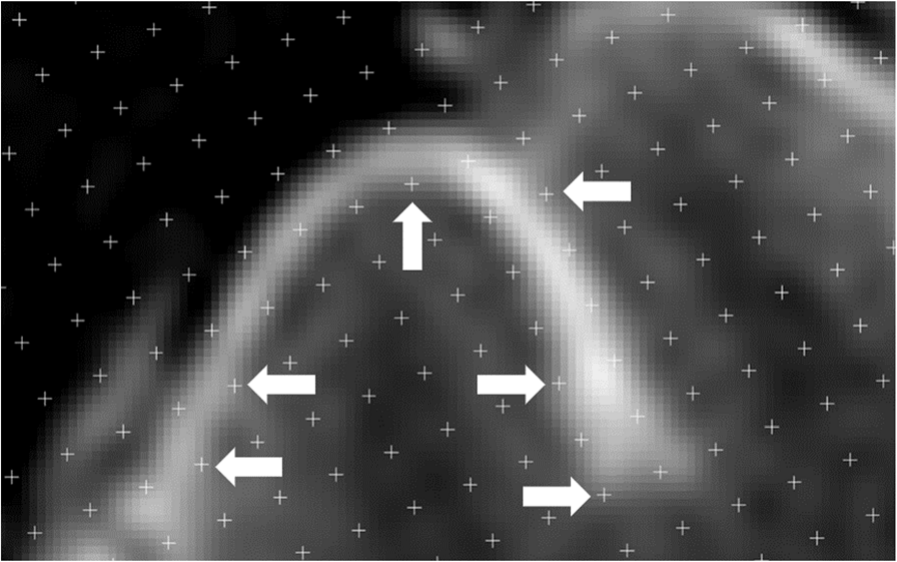
Example MRI brain image analysed in this paper highlighting the pixellation and subjective white to black colour gradient. White arrows highlight some cross-points where potentially difficult and subjective decisions were made

## Discussion

To the author’s knowledge this is the first study to use Cavalieri’s stereological principle to estimate brain volume in dogs with IE. In this study, no differences in FB, SAS and LV volume were found between dogs with IE and controls. Associations between clinical history and brain volume were found within the IE group but, as will be discussed further below, these results require further research to substantiate.

### Effect of IE on FB, SAS and LV volume

This study found no statistically significant differences in FB, SAS and LV volume in dogs with IE compared to controls. Whilst many studies have focused on LV volume, we believe this is the first study to report FB and SAS volumes in dogs with IE compared to controls. Unfortunately, “negative” results such as these can be easily dismissed, but these results further support the need for a rigorous diagnosis of exclusion for dogs with IE, given that there are still no diagnostic biomarkers specific to IE. Furthermore, volumetric studies are important in veterinary medicine to establish a database of “normal” volumes for various breeds and disorders.

Most volumetric studies in dogs with IE compared to controls focus on total LV volume (as in this study) or on left/right LV asymmetry [13, 21, 24, 27, 28] reporting mixed findings. This study contributes to this existing body of research, perhaps strengthening the conclusion that there is no difference between total LV volume in dogs with IE compared to controls. Similarly, volumetric studies in people with epilepsy have reported various additional structural abnormalities [4–9, 11, 13], though a unanimous hypothesis on the cause of these changes has not been reached.

Historical veterinary studies [20, 25, 30] have suggested normalising the volume of the LV to the overall brain volume, especially when comparing different breeds. Using the data acquired in this study we expressed FB volume as a relative value and expressed LV volume as a percentage of FB volume, but results did not change the conclusion already made. Importantly, the body weights of dogs included in this study were not significantly different between IE and control groups, as Schmidt, et al. [31] have shown that brain volume is significantly affected by body weight in dogs. Though, it is worth mentioning that body condition score was not taken into account by either Schmidt, et al. [31] or the present study.

Pilegaard, et al. [32] found that breeds with a wide (laterolateral) but shallow (rostrocaudal) skull shape had a smaller cortical volume to ventricular volume ratio. Similarly, Bakici, et al. (2019) [33] used the Cavalieri method to compare brain fraction volumes, including lateral ventricles, of brachycephalic and mesocephalic breed types and found no significant difference, although the aforementioned authors’ findings should be interpreted with caution because, in stereology, volume fractions are normally limited and sometimes inconclusive and cannot be directly compared with more accurate global volumes as the ones we estimated in our study. Of the eight breeds included in this study, all are mesocephalic aside the Boxer, which was not measured due to low numbers. Therefore, the skull type of the dogs included is unlikely to have skewed the data as all dogs were a similar size and skull shape. Additional analysis to confirm this assumption by comparing FB, SAS and LV volume for Border Collies, Labradors, German Shepherd Dogs and Golden Retrievers with IE to controls separately by breed showed no significant differences in volumes for the FB, SAS or LV in any of the breeds, which further supports the negative findings reported.

### Effect of time since the onset of IE

The time since the onset of IE was moderately-positively correlated to volume of the LV, but this finding was only a statistical trend. Figure 2 suggests this result is largely driven by two dogs, a Labrador Retriever and Golden Retriever, with large LVs where onset of IE was more than 1000 days prior to MRI. To assess this, a multivariate analysis of variance (MANOVA) was carried out, which showed no significant interactions between breed, LV volumes and time since seizure onset. Given the dataset was small, the dataset was copied once to double it. This second analysis with the doubled dataset showed a significant relationship between breed, LV volume and time since seizure onset (*p* = 0.005), of which the significant contributing interaction was breed and LV volume (*p* = 0.001). In this instance breed explained 34.8% of the LV volume difference. A follow-up one-way ANOVA showed Golden Retrievers to have significantly larger LV volumes than Border Collies and German Shepherd Dogs. Based on these rudimentary results, the authors would suggest careful selection and control of dog breed in the further longitudinal analysis of LV volume in dogs with IE recommended above. Though tempting to include many different dog breeds in a study to broaden the application of findings, the large conformational diversity of dogs may prohibit this by introducing avoidable variability.

A relationship between these variables in dogs with IE is biologically plausible; in a study on rat models of SE [34], it was found that LV volume increased over time. However, in dogs with IE, Kuwabara, et al. [28] reported no association between the hippocampi asymmetry ratio and the time since onset of seizures.

It has previously been reported that LV dilatation is positively correlated to increasing age in dogs [35–37] and people [38] and, therefore, age could be a confounding variable, in addition to the breed factors already discussed. Furthermore, Noh, et al. [39] have recently shown brain atrophy in a group of dogs over 9 years of age with cognitive dysfunction and, therefore, the effect of age on this population must be considered. The maximum age of dogs in this analysis was 79 months (6 years and 7 months), which would be considered middle-age. However, a previous study investigating canine cognitive dysfunction (CCD) in dogs with IE found onset of CCD-related clinical signs to be earlier than expected [40]. Without the support of MRI scans we cannot know if this is due to structural changes, but it would be an excellent route for further study. Dogs less than 6 months of age were not included, per the lower limit in the IVEFT guidelines for diagnosis of IE [3].

Though strong conclusions cannot be made, this study suggests that LV volume change over time could be a valuable future research hypothesis to test in a large veterinary longitudinal MRI study.

### Cluster seizures and status Epilepticus

Whilst it is likely that dogs with CS or SE have been included in groups of dogs that have IE during volumetric studies, to the authors’ knowledge they have never been assessed as a separate entity. The FBs of dogs with SE were found to be significantly larger than controls, which was surprising and did not match clinical suspicions held prior to the study. Further investigation showed poor breed distribution in these two groups despite systematic and uniform random sampling (SURS); the SE group contained only Labradors and Golden Retrievers, whilst the control group only contained one of these dog breeds. Additionally, an outlier in the control group shows a Border Collie with a particularly small forebrain. It is likely that breed distribution is a factor in this result too and, therefore, we cannot confidently conclude that the FB is larger in dogs with IE who experience SE. Despite following standard methodology for stereological studies with regards to group sizes, we recommend breed-specific or larger breed-diverse analysis is carried out in future to identify significant trends should they exist..

Despite our results not aligning with expectations and likely breed-bias of our results, post-ictal changes in brain structure following a seizure or SE have been reported in people [41] and dogs [3]. In human medicine it is known that prolonged seizure activity causes neuronal death [42], and Hocker, et al. [43] demonstrated that cortical and subcortical atrophy occurs in people who experience super-refractory SE (SE that recurs or continues for more than 24 h despite the use of anaesthesia). Newey, et al. [44] reported one case where global cerebral atrophy occurred progressively in a patient with super-refractory SE.

### Use of the Cavalieri principle

The Cavalieri principle was easy to use after a period of training and practice to familiarise the observer with the software, however, it was a time-consuming process with each brain taking 60–90 min to measure. The high density of cross-point markers was necessary to account for the relatively small volume of the LV so that a sufficient number of points could be counted to ensure an acceptable CE, meaning that thousands of cross-points were counted in larger areas such as the FB. If an observer was only interested in one anatomical structure then the density of the cross-points could be adjusted to account for this, thus speeding up the process.

Two studies have detailed the accuracy of the Cavalieri principle using MRI scans compared to the actual brain post-mortem; Furlong, et al. [45] found poor agreement between MRI and pathological volumetric analysis of the cortical and subcortical volume and thickness of the cerebral cortex in human brains post-mortem, and they concluded that this was likely due to the poor resolution provided by the 3 T MRI used. A similar study [46] used the Cavalieri principle to compare pig brain volumes on 3 T MRI under anaesthesia and on physical sections following immediate euthanasia. They found no statistically significant differences between estimation methods in all areas except the basal nuclei compartment. Whilst this is not considered an appropriate statistical measure for agreement, the authors reported mean differences of 3–5% for the total brain volume, which are acceptable, but up to 9–11% for the other compartments, indicating a less satisfactory level of agreement than the t-tests suggest. Given this evidence it could be that smaller and more subtly defined areas measured using the Cavalieri principle on MRI do not agree well with true physical sections due to poor resolution of the anatomical structure represented in the MRI or that a finer grid needs to be used. The use of contrast solutions (e.g. gadolinium) during MRI could be investigated as a potential way to improve the resolution required for the better visualisation of the anatomical area of interest.

One challenge of using the Cavalieri principle with digital images can be seen in Fig. 4. Due to the nature of MRI images representing the average of a slice thickness, potential for a partial volume effect, the use of a fine saturation gradient of white, grey and black, and the need to magnify an image for accuracy thus creating a very pixelated image, means measurements can have a certain degree of subjectivity.

**Fig. 5:**
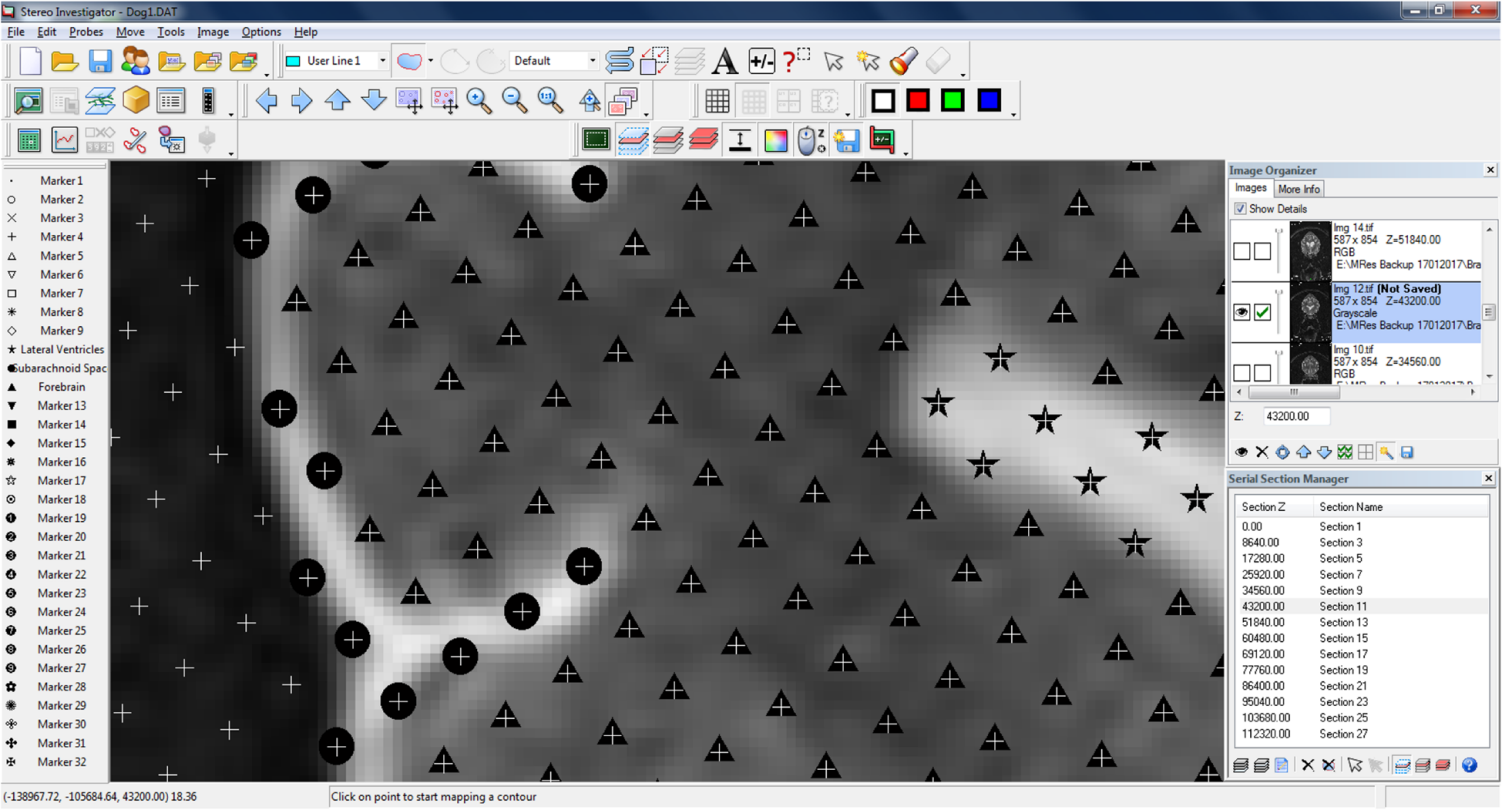
Transverse section of the brain seen in MRI: a plethora of markers can be used for the delineation of different brain anatomical structures. The triangle (▲), circle (●) and star (★) demark the forebrain, subarachnoid space and lateral ventricles, respectively

### Limitations

The presence of two dogs with mildly increased protein in their CSF presumed due to recent seizure activity could have biased the study. The IVEFT recommend repeating these tests 16 and six weeks later, respectively [3], however, in veterinary medicine this is unlikely to occur because care is privately funded. For further support of IE diagnosis follow-up clinical examination can be repeated 1–3 years later to confirm no development of interictal neurological deficits. Due to the referral population of these dogs the mean days since seizure was well under this recommendation.

The MRI scanners used in this study were 1.5 T, therefore, not comparable to 3 T and above scanners available in human medicine. It is possible that with a 3 T scanner, differences could be found or that longer scans on the 1.5 T scanners would be preferable for volumetric analysis. A study comparing image quality, resolution and contrast of MRI images of the canine brain taken on a 3 T and 7 T MRI scanner found that both had their preferred uses and that some structures were better visualised on 3 T and some on 7 T [47]. The authors suspected that the use of 7 T MRI could help in the explanation of certain pathology in brain disorders such as epilepsy, but access to these powerful magnets is very limited in a veterinary clinical or research environment, which inhibits further research in this area. T2 TRA sequences were chosen because they offered good tissue contrast and were commonly used across patients with epilepsy and controls, and across both hospital sites. This decision has potential to affect the measurement process and should be taken into consideration when comparing to the findings of existing or future research.

## Conclusions

This research contributes to our current understanding of IE diagnosis in dogs, but also emphasises the need for further work in both healthy and pathological canine neuroanatomy, and the need for longitudinal investigation. No significant differences were found for FB, SAS or LV volume between dogs with IE and controls and, for now, IE remains a diagnosis of exclusion with no specific neuroanatomical biomarkers identified. Strong conclusions regarding association between LV volume and time since seizure onset or FB, SAS and LV volume in dogs with CS or SE compared to IE-controls cannot be made. The authors would advise strong controls for breed variation in future studies or active work on “normal ranges” for specific breeds so that these can be accounted for. Finally, the Cavalieri principle was an easy and effective estimation of FB, SAS and LV volumes on MRI images and shows promise for future research but may be too time-intensive for use in clinical practice.

## Materials and methods

MRI scans were identified from the databases of two neurology referral hospitals with 1.5 T MRI scanners with at least T2-weighted transverse imaging of the entire brain, a common scan type in veterinary IE protocols. Eight breeds of dog previously identified as being at an increased odds of having IE compared to cross-breeds were included: the Golden Retriever, Labrador Retriever, Cocker Spaniel, Border terrier, German Shepherd dog, Parson Jack Russell terrier, Boxer, and Border Collie [2]. One database was searched manually and the other using VetCompass.^1^ Dogs with IE were required to have at least a Tier I confidence level diagnosis [3] for inclusion. Controls were identified from dogs found to have an unremarkable brain on MRI (for reasons other than seizures) and following thorough examination of their clinical history. All dogs were assessed and diagnosed by a Diplomat of the European College of Veterinary Neurology (ECVN) or ECVN Resident. The term “unremarkable brain on MRI” means that it is perceived to be “normal” and that no anatomical brain abnormalities could be detected – it is similar to the human medical term, “MRI-negative”.

IE and control dogs of each breed were SURS from the resulting population of dogs for volumetric analysis. Breeds with three or less dogs in either IE or control groups were excluded from further analyses. The volume of the FB, SAS and LV (total left and right) were estimated by one blinded observer (FW) using the Cavalieri principle on the T2-weighted transverse brain MRI.

### Cavalieri’s principle

The Cavalieri principle is an unbiased and efficient design-based stereological method used to accurately estimate the volume of three-dimensional structures using a series of SURS representative two-dimensional slices [48]. The Cavalieri principle uses a robust 3D stochastic sampling procedure, which means that a sample size of 5–10 subjects can be used effectively to detect inter-group differences [49, 50]. The estimated volume is calculated as follows,

$$ \hat{V}={A}_p{m}^{\prime}\overline{t}\left[\sum \limits_{i=1}^n{P}_i\right] $$

Where *A*_*p*_ is the area associated with a point, *m*^′^ is the section evaluation interval, $\overline{t}$ is the mean section cut thickness and *P*_*i*_ is the number of points counted on the grid. The associated coefficient of error (CE) is calculated as follows,

$$ CE=\frac{\sqrt{TotalVar}}{\sum_{i=1}^n{P}_i} $$

Where *TotalVar* is the total variance of the estimated volumes, *n* is the number of sections and *P*_*i*_ is the number of points counted on the grid. In this study all comparisons were made with a minimum group size of *n* = 5.

### Estimation procedure

**Fig. 6:**
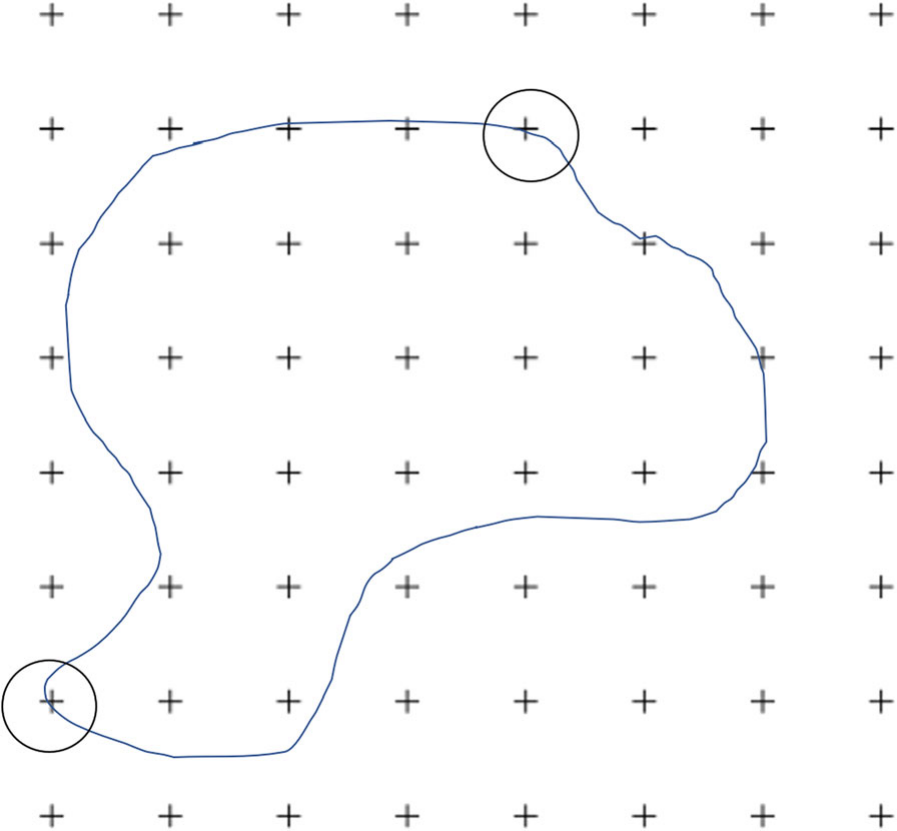
Cross-points are counted when the top-right corner is within the structure of interest. For example, the circled cross-point in the bottom left of this figure is counted, but the circled cross-point in the top-right of this figure is not

The stereological software StereoInvestigator^2^ was used to produce all final estimations. A minimum of 10 slices throughout a whole structure is recommended to decrease the CE and, therefore, MRI sequences with 20–29 and 30–39 slices were imported with a slice interval of 2 and 3, respectively. MRI slices were imported, and slice thickness was manually entered into the programme per the technical details of each scan. A 1.5mm^2^ point grid was chosen so that the smallest area of interest (LV) would contain a great enough number of points to achieve a low CE. The area associated with each point was informed by the software. The grid was superimposed over each slice at an angle randomly assigned by the software. Starting at the most rostral slice, points within an area of interest were highlighted using a different marker for each of the three structures, as shown in Fig. 5. Cross-points were counted when the top-right corner was within the structure of interest, as pictured in Fig. 6.

### Measurement reliability

A training period to ensure accurate identification of desired anatomical structures and sufficient point grid density was carried out, followed by a pilot study of six dogs to check that the observer (FW, a Registered Veterinary Nurse) took measurements as accurately as a final-year ECVN Resident (AT). The training and pilot study were carried out manually, without the assistance of StereoInvestigator. An acetate page with a 1cm^2^ (for FB) and 2.5mm^2^ (for LV and SAS) cross-point grid was printed and attached to a computer screen displaying MRI slices at 1:1 magnification. Points were counted, manually recorded and volumes estimated. All other previously described methodology was upheld.

### Statistical analyses

The data that support the findings of this study are openly available in Figshare at 10.5522/04/12578264.

Patient data was recorded on Microsoft Excel^3^ and exported to SPSS 23^4^ to generate descriptive statistics. Raw stereological data was exported from StereoInvestigator to Microsoft Excel and exported to MiniTab 17^5^ for inferential analyses. For the pilot study, mean volumes and the confidence intervals were reported, and for final measurements CE was used to express precision of the estimate [50]. Mean and standard deviation (SD) were reported for descriptive data regardless of data distribution and, for final volumetric data, the group mean was given alongside the coefficient of variance (CV) and the mean CE. CV represents the CE derived from the methodology and the genetic CV derived from the inherent biological variability of the animals. Inter-rater agreement was tested by calculating the intra-class coefficient (ICC) with a two-way mixed effects model for absolute agreement.

Data were tested for normality and homoscedasticity. Independent Samples t-tests, Kruskal-Wallis tests and Mood’s Median tests were used to compare groups, as appropriate. Where significant differences were found, Tukey’s Pairwise Comparisons were used for post-hoc analyses. The relationships between two continuous variables were tested with Pearson’s product-moment correlation. Statistical differences were considered significant at *p* < 0.05.

### Ethics statement

Research was approved by the Royal Veterinary College Clinical Research Ethical Review Board (URN M2015 0053).

## Data Availability

The datasets generated and/or analysed during the current study are available in the University College London Figshare repository, 10.5522/04/12578264.

## Abbreviations

AED: Anti-epileptic drug
BAST: Bile acid stimulation test
CE: Coefficient of error
CS: Cluster seizures
CSF: Cerebrospinal fluid
CV: Coefficient of variance
ECVN: European College of Veterinary Neurology
FB: Forebrain
IE: Idiopathic epilepsy
IVETF: International Veterinary Epilepsy Task Force
LV: Lateral ventricles
MRI: Magnetic resonance imaging
SAS: Subarachnoid space
SE: Status epilepticus
SURS: Systematic uniform random sampling

## Footnotes

1 www.vetcompass.org

2 Stereo Investigator, MBF BioScience

3 Microsoft Excel for Mac 2016

4 IBM SPSS Version 23

5 MiniTab®17
